# Identification of Copy Number Variations and Genetic Diversity in Italian Insular Sheep Breeds

**DOI:** 10.3390/ani12020217

**Published:** 2022-01-17

**Authors:** Rosalia Di Gerlando, Salvatore Mastrangelo, Marco Tolone, Ilaria Rizzuto, Anna Maria Sutera, Angelo Moscarelli, Baldassare Portolano, Maria Teresa Sardina

**Affiliations:** 1 Dipartimento Scienze Agrarie, Alimentari e Forestali, University of Palermo, 90128 Palermo, Italy; salvatore.mastrangelo@unipa.it (S.M.); marco.tolone@unipa.it (M.T.); ilariz91@gmail.com (I.R.); angelo.moscarelli@unipa.it (A.M.); baldassare.portolano@unipa.it (B.P.); mariateresa.sardina@unipa.it (M.T.S.); 2 Dipartimento Scienze Veterinarie, University of Messina, 98168 Messina, Italy; asutera@unime.it

**Keywords:** genetic diversity, copy number variations, sheep breed

## Abstract

**Simple Summary:**

The aim of this work is to identify CNVs and perform a CNV-based population genetics analysis in five Italian sheep breeds (Barbaresca, Comisana, Pinzirita, Sarda, and Valle del Belìce). We identified 10,207 CNVs and 365 CNV regions (CNVRs). The different approaches used to disclose the genetic relationship showed that the five breeds tend to cluster in distinct groups. Gene enrichment was described for the 37 CNVRs selected considering the top 10%. Gene Ontology analysis showed that several of these genes are involved in lipid metabolism, immune response, and the olfactory pathway. CNVs represent valuable molecular resources to provide useful information for separating the population.

**Abstract:**

Copy number variants (CNVs) are one of the major contributors to genetic diversity and phenotypic variation in livestock. The aim of this work is to identify CNVs and perform, for the first time, a CNV-based population genetics analysis with five Italian sheep breeds (Barbaresca, Comisana, Pinzirita, Sarda, and Valle del Belìce). We identified 10,207 CNVs with an average length of 1.81 Mb. The breeds showed similar mean numbers of CNVs, ranging from 20 (Sarda) to 27 (Comisana). A total of 365 CNV regions (CNVRs) were determined. The length of the CNVRs varied among breeds from 2.4 Mb to 124.1 Mb. The highest number of shared CNVRs was between Comisana and Pinzirita, and only one CNVR was shared among all breeds. Our results indicated that segregating CNVs expresses a certain degree of diversity across all breeds. Despite the low/moderate genetic differentiation among breeds, the different approaches used to disclose the genetic relationship showed that the five breeds tend to cluster in distinct groups, similar to the previous studies based on single-nucleotide polymorphism markers. Gene enrichment was described for the 37 CNVRs selected, considering the top 10%. Out of 181 total genes, 67 were uncharacterized loci. Gene Ontology analysis showed that several of these genes are involved in lipid metabolism, immune response, and the olfactory pathway. Our results corroborated previous studies and showed that CNVs represent valuable molecular resources for providing useful information for separating the population and could be further used to explore the function and evolutionary aspect of sheep genome.

## 1. Introduction

Copy number variants are DNA segments widely dispersed in mammalian genomes, including deletions, duplications, and insertions, ranging from 1 kb to several Mb, that vary compared with a reference genome [1]. According to the number of copies of the segment, CNVs can be classified in deletions (or losses) and duplications (or gains). CNVs involving large genomic regions can affect the gene structure and gene dosage, which, in turn, has an impact on gene expression. In fact, these structural variations are one of the major contributors to genetic diversity and phenotypic variation in many species, including sheep [2,3,4,5], cattle [6,7,8,9], pigs [10], and dogs [11,12,13]. Although CNVs have been mapped in most species, their use as markers for population genetics studies has been proposed in few livestock species, such as cattle [8,14,15,16], sheep [17], goat [18], and turkey [19].

Examining genetic diversity in local breeds is important because it could help evaluate the evolutionary processes that lead to divergence and differences between and within them. Therefore, understanding the multiple components of functional breed diversity have important implication for breed management and genetic improvement practices, especially in breeds that are locally adapted and have not undergone intense artificial selection [6].

As microsatellites and single-nucleotide polymorphisms (SNPs) have been used to examine population structures and genetic diversity in order to obtain information on origin, history, and adaptation of breeds [20,21], the use of CNVs could be relevant. Several authors found CNVRs harboring annotated genes related to expressed phenotypes caused by the specific evolutionary history of the populations [8,15,19,22,23]. Moreover, due to CNV’s less-known linkage disequilibrium (LD) patterns, CNV-based population genomics results could offer additional new insights for functional and evolutionary studies in livestock [18].

The aim of this work was to identify CNVs and perform, for the first time, a CNV-based population genetics analysis of five insular Italian sheep breeds. This study could offer new insights into the genomic architecture of local sheep and facilitate our understanding of the evolution and subsequent selection within the sheep genome.

## 2. Materials and Methods

Blood samples were collected by private and official veterinarians of the local health authorities in the contest of sanitary programs. All the procedures were in agreement with the recommendations of the European Union Directive 2010/63/EU, to ensure appropriate animal care.

### 2.1. Sampling and Genotyping

A total of 667 individuals from five Italian sheep breeds—Barbaresca (BARB, n = 30), Comisana (COM, n = 72), Pinzirita (PIN, n = 77), Sarda (SAR, n = 30), and Valle del Belìce (VDB, n = 468) —were sampled for this study. These breeds present differences in both phenotypic (coat color, body size, and weight) and production traits (e.g., milk production), and show excellent adaptability to the local environments [24,25]. The COM, PIN, SAR, and VDB breeds are reared for milk production, while BARB is a dual-purpose breed. In particular, the PIN is an ancient local breed; due to its good adaptive traits and hardiness, it is reared on farms located in marginal areas for milk production, representing an important genetic resource for present and future needs [26]. The COM is one of the most important breeds mostly reared in Central and Southern Italy. The breed is valued for its high milk yield, it is generally completely white in color, and its face is brick-red with a white frontal stripe. The SAR—a hornless breed with white fleece, selected since the 1930s for milk production—represents nearly the totality of the insular population with about 3 million sheep reared [27,28]. The VDB is the most important dairy sheep in Sicily; it is likely that this breed derives from the PIN breed, to which it is similar for the horned trait in males, crossed with the COM breed, to which it is similar for coat color (i.e., white with red head) and milk production. Subsequently, the cross between these two breeds was likely crossed with the SAR breed [24,25,26,27,28,29]. The BARB is a dual-purpose ancient sheep with a long and pendulous tail, reared in a very restricted area in central Sicily under a semi-extensive farming system. The breed seems to originate from crosses between Tunisian Barbary sheep from North Africa and the PIN breed and is, at present, highly endangered [30]. In Italy, the BARB together with the Laticauda are the only two fat-tail sheep breeds [31].

Genotyping was performed using the Illumina OvineSNP50K BeadChip v2 array containing 54,241 SNPs. The positions of the SNPs on the chromosomes were determined from the ovine Oar_v3.1 genome assembly. All the 667 genotyped individuals passed the quality control criteria of call rate > 98%. Unmapped SNPs and sex chromosomes were excluded from the analysis, leaving 52,413 markers for CNV mapping.

### 2.2. CNV and CNVR Detection

The optimal segmenting (CNAM) module of SVS 8.7.0 (Golden Helix Inc., Bozeman, MT, USA; www.goldenhelix.com (accessed on 3 July 2021)) was used to identify CNVs using the univariate approach that segments each sample independently [32]. We imported the Log R Ratio (LRR) values for each SNP from GenomeStudio 2.0 software (Illumina Inc., San Diego, CA, USA) into SVS 8.7.0. Quality assurance of the LRR data and filtering of outlier samples were performed using SVS software, as described by Pinto et al. [33]. Individuals were screened for their GC content, which is correlated to long-range waviness of LRR values. Outlying samples were detected by the SVS 8.7.0 for waviness [34] and those identified were deleted.

The CNVRs were determined by aggregating the overlapping CNVs identified in at least two detections across all samples within each breed [35]. Overlapping was identified with the BEDTools software [36]. CNVRs were treated as individual loci and only those identified, within each breed, in at least five individuals were used to reduce false positives within the dataset. The VENN diagram web tool (https://bioinformatics.psb.ugent.be/webtools/Venn/ (accessed on 3 July 2021)) was used to create a Venn diagram showing the overlap between CNVRs identified in different breeds.

### 2.3. Comparison of CNVRs between Breeds

Three input files were constructed and applied to analyze the genetic relationships among breeds [15]. The first contained 427 animals with presence (“1”) or absence (“0”) of each CNVR loci (n = 365). The second dataset included presence/absence data of the CNVR loci in each of the five sheep breeds, while the third dataset contained information on the CNVR loci frequencies in each breed. Different approaches were used in order to disclose population structure and diversification of these five breeds. The GenAlEx 6.5 software [37] was used to calculate the pairwise population PhiPT values (analogous to Wrights’ Fst index) by mean of an AMOVA using 9999 permutations [15]. The PAST 3.22 software [38] was used to perform the Principal Component Analysis (PCA) of pairwise individual genetic distances, and a Hierarchical Clustering Analysis (HCA) using Euclidian distance measure and UPGMA as clustering method. Moreover, the Heatmap analysis using the top 10% of CNVRs based on their variance among the five breeds was conducted.

### 2.4. Gene Content and Functional Annotation

The gene content of CNVRs was assessed based on Oar_v3.1 in the Genome Data Viewer browser from the US National Center for Biotechnology Information (NCBI) database.

Gene ontology analysis was performed with PANTHER Classification System v16.0 [39] using Bonferroni correction at a significance level of 0.05. To investigate the biological function and phenotypes that are known to be affected by each identified gene, we conducted a comprehensive literature search, including information from other species.

## 3. Results

### 3.1. CNVs and CNVRs Detection

**Figure 1:**
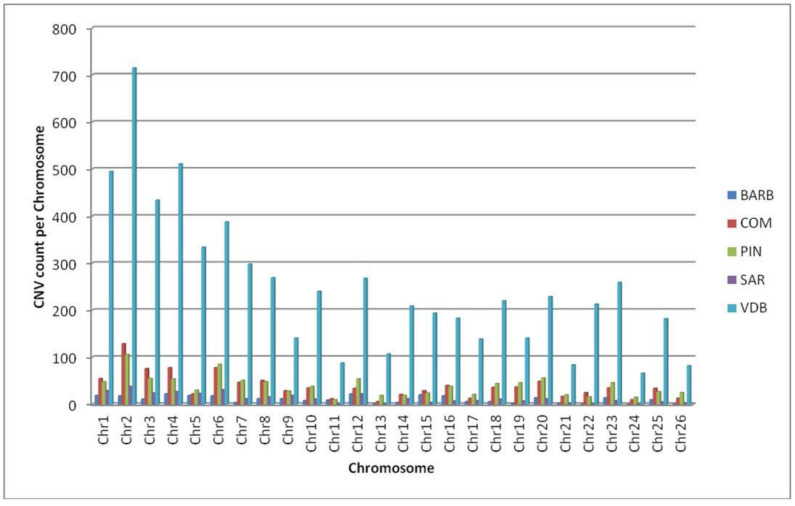
CNV count for 26 autosomes across five breeds. Barbaresca (BARB), Comisana (COM), Pinzirita (PIN), Sarda (SAR), and Valle del Belìce (VDB).

The quality control performed with SVS 8.7.0 allowed for the identification of 240 outlier individuals. Therefore, the final dataset used for analyses comprised a total of 427 individuals (BARB (n = 19), COM (n = 43), PIN (n = 47), SAR (n = 24), and VDB (n = 294)). The total number of CNVs (Table S1) called across the 26 autosomal chromosomes was 10,207 and varied in terms of number and size among the breeds (Table 1 and Figure 1).

The breeds showed similar mean numbers of CNVs, ranging from 20 (SAR) to 27 (COM). BARB showed lowest mean length, while VDB had the longest one. Among identified CNVs, 8052 were deletions (loss) and 2155 were duplications (gain). CNVs ranged from 13,128 bp to 14.99 Mb in size with an average length of 1.81 Mb.

The highest number of CNVs (n = 715) was found on chromosome 2 in the VDB, while no CNV was identified on chromosomes 13, 22, 24, and 26 in the BARB and SAR breeds.

Descriptive statistics of CNVRs identified in the five sheep breeds are reported in Table 2. A total of 1240 CNVRs were obtained across all breeds with 960 losses and 280 gains.

**Figure 2:**
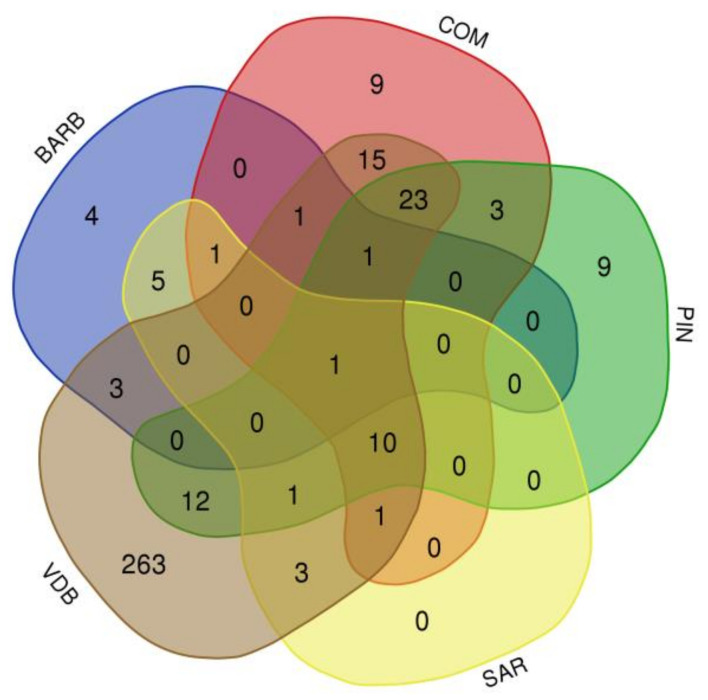
Venn diagram representing common and unique CNVRs found among the five breeds. Barbaresca (BARB), Comisana (COM), Pinzirita (PIN), Sarda (SAR), and Valle del Belìce (VDB).

A total of 365 CNVRs (Table S2) were determined by aggregating the overlapping CNVs identified across all samples and present in at least five individuals of the same breed. In particular, 16, 67, 60, 23, and 349 CNVRs were identified in the BARB, COM, PIN, SAR, and VDB breeds, respectively. The length of the CNVRs varied among breeds from 2.4 Mb (in BARB) to 124.1 Mb (in VDB). A comparison of the CNVRs among breeds is showed in the Venn diagram (Figure 2). The highest number of shared CNVRs was between COM and PIN (n = 23), followed by the ones shared between COM and VDB (n = 15). Only one CNVR was shared among all breeds.

### 3.2. CNVR Genetic Diversity Analyses

**Figure 3:**
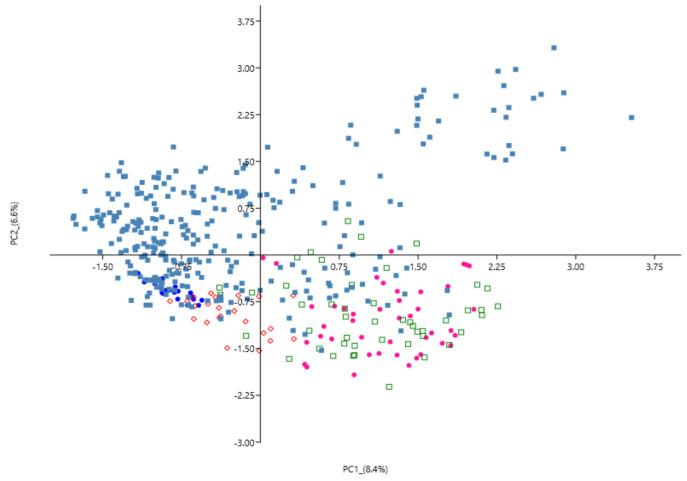
Principal components (PC) analysis for the genetic differentiations among sheep breeds using PC1 and PC2. Barbaresca (BARB ∙), Comisana (COM ∙), Pinzirita (PIN ▫), Sarda (SAR ◊), and Valle del Belìce (VDB ▪).

**Figure 4:**
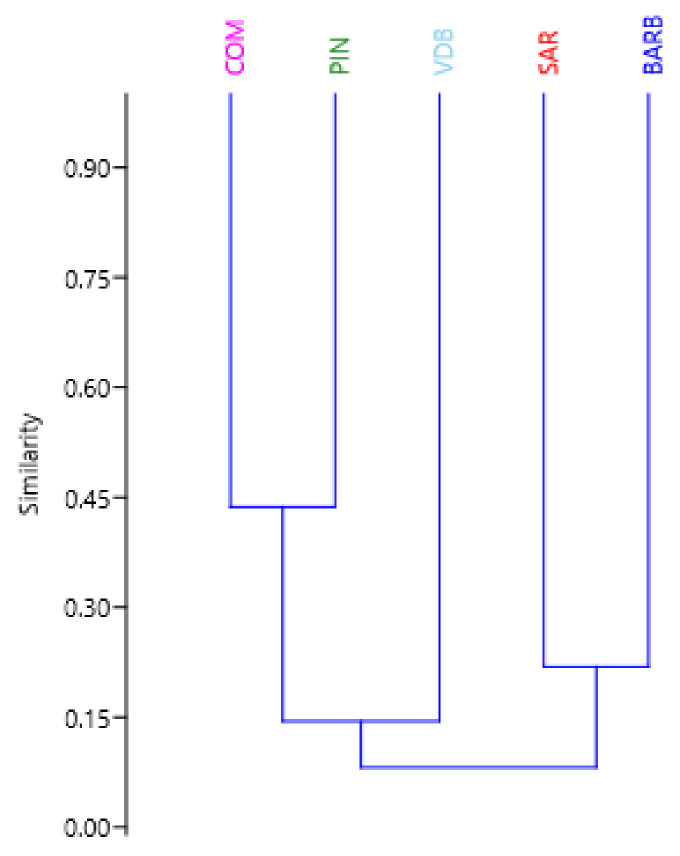
Dendrogram cluster analysis based on frequency of CNVRs in the five sheep breeds. Barbaresca (BARB), Comisana (COM), Pinzirita (PIN), Sarda (SAR), and Valle del Belìce (VDB).

**Figure 5:**
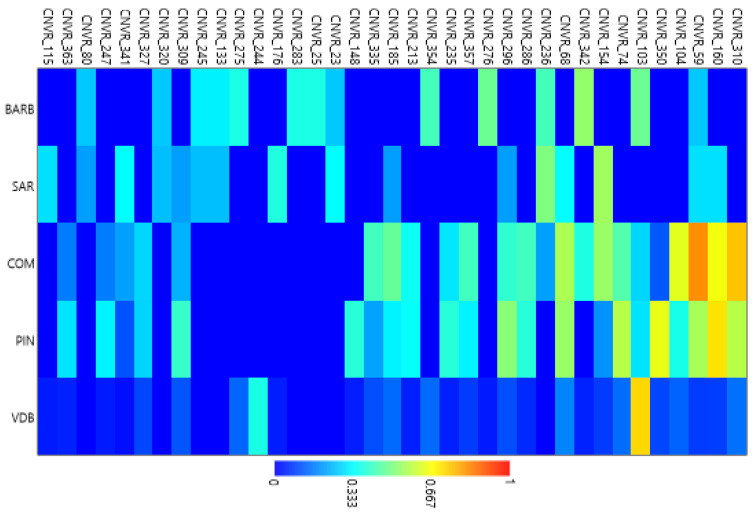
Heatmap analysis based on hierarchical cluster using top 10% of CNVRs in the five sheep breeds. Barbaresca (BARB), Comisana (COM), Pinzirita (PIN), Sarda (SAR), and Valle del Belìce (VDB).

In the PCA analysis (Figure 3), the first two components (PC1 and PC2) explained 8.4% and 6.6% of the total variance, respectively. The individuals of BARB had the most compact clustering. The SAR breed was positioned in the same area. The VDB individuals showed a more spread cluster. Finally, COM and PIN were positioned a little further from the other breeds (bottom right area of the Figure 3). A cluster dendrogram per breed is depicted in Figure 4. The results show two distinct branches separating the three dairy Sicilian sheep breeds (COM, PIN, and VDB) from the SAR and the dual purpose BARB. Finally, we performed a cluster heatmap analysis using the top 10% of CNVRs (Table S3) considering their variances among the five breeds (Figure 5). This final result clearly arranged the breeds according to the above reported analyses.

To evaluate CNVRs’ contribution to population differentiation, we estimate the pairwise PhiPT genetic distances among the five breeds (Table 3). The Analysis of Molecular Variance (AMOVA) based on PhiPT values indicated that most of the genetic diversity occurred within populations (88%) while the variability among populations contributed 12% (Table S4). The value of PhiPT varies between 0 (no population differentiation) and 1 (full differentiation). The PhiPT distances between breeds are statistically significant with *p*-value < 0.0001 based on 9999 permutations. The highest value was estimated between BARB and PIN, and the lowest one between PIN and COM.

### 3.3. Gene Enrichment and Functional Annotations of CNVRs

Gene enrichment was described for the 37 CNVRs selected considering the top 10%, and 29 of them encompassing genes. The CNVR_309 located on chromosome 19 was the one that contained the greatest number of genes (n = 49) (Table S3). Out of 181 total genes, 67 were uncharacterized loci. Only the CNVR_39, located on chromosome 2 and containing the*LRP1B* (Low-Density Lipoprotein Receptor-Related Protein 1B) gene, was common to all breeds.

Based on PANTHER analysis, the enriched GO terms included biological processes (biological regulation, cellular and metabolic processes, response to stimulus, and immune system process), molecular function (binding, catalytic activity molecular function regulator, and transporter activity), and cellular component terms (cellular anatomical entity, intracellular, and protein-containing complex) (Table 4).

## 4. Discussion

In general, several studies of genetic diversity have been conducted in Italian sheep breeds [40,41], particularly in insular breeds, using molecular markers as microsatellites [24] and SNPs [30], while knowledge is limited regarding their characterization and genetic variation using CNVs. In this work, we studied the genomic variability of five Italian sheep breeds based on CNVs and CNVRs information.

The number of CNVs and CNVRs identified in this study was not directly comparable with other previously reported papers due to differences in the used algorithms, technologies, filter criteria, and numbers of tested samples and breeds [42]. Although the use of SNP arrays is, nowadays, a standard method, they vary in density and number of markers, ranging from 50K [2,3,4] to 600K [43,44,45]; for example, Ma et al. [44], analyzing 48 Chinese sheep with the 600K SNP array, identified a higher number of CNVRs (1296) with a smaller size (about 96 Kb) compared with ours. Furthermore, Ma et al. [3] reported 111 CNVRs from 160 Chinese sheep with an average size of 123.78 Kb using the 50K SNP array. The amount of “loss” CNVRs results prevalent respect to the “gain” ones, in agreement with previous studies [3,4,46]. The detected CNVRs are probably underestimated due to SNP density on the 50K array. This drawback could probably be avoided by using Ovine HD SNPs, which could provide higher resolution and sensitivity for CNV detection and population analysis than the low-density SNP array. Thus, the density of array is an important factor that affects the CNV discovery and, therefore, their use in population genetic analyses [17].

Different approaches were used to disclose population structure and differentiation among the five breeds; in general, the CNVRs characterization and genetic diversity analyses demonstrate that the five breeds tend to cluster in distinct groups.

The genetic distances obtained by CNVRs using PhiPT values (Table 3) indicated greater differentiation between BARB and PIN, and a lower distance between COM and PIN. These last two breeds were also grouped together for the higher number of shared CNVRs (n = 23) (Figure 2). Similar results for COM and PIN were found, in previous studies, using microsatellite markers [24], SNP array [30], and whole-genome resequencing data [47].

PCAs generated from identified CNVRs showed less variation among the five breeds than those based on thousands of SNPs [25,30]. For example, CNVRs cannot distinguish the BARB from the other Sicilian breeds and showed shared area between COM and PIN. Similar results were also reported in cattle [48] and horse [49]. Bickhart et al. [50] performed a MDS analysis based on CNVs’ genotypes and compared it with the plot based on SNPs in a study on taurine and zebuine cattle, showing that the separation and clustering of the taurine using CNVs were not superior to those based on SNPs; the authors suggested that CNV genotyping still has room for improvement. In fact, compared with SNPs, CNVs suffer from small sampling size and difficulty to genotype, making it difficult to use them for fine clustering, especially within a group [48].

Long-term adaptation to different environmental conditions or different selection schemes increases the presence of specific copies of genes and, therefore, variation in CNVs among breeds [19,51]. Our results showed low level of differentiation among the five breeds due to breeding practices and similar environmental conditions, gene flow, and shared ancestral components. All these factors led to an increase in shared CNVRs among breeds. Dendrogram per breed (Figure 4) showed two main groups, one with VDB, COM, and PIN (with COM and PIN closer), the other group formed by BARB and SAR. This result is due to the highest number on CNVRs shared by VDB, COM, and PIN than with BARB and SAR, in which they lacked. Therefore, the obtained results suggest that CNVs/CNVRs represent a valuable molecular resource to provide good information for separating the populations among them and could be further used for exploring the function and evolutionary aspect of sheep genome.

Out of the 37 genomic regions (Table S3), 29 CNVs encompassed 181 genes, some of which were related to lipid metabolism, immune response, olfactory receptor, and different biological process.

The proteins of the *LRP1B* gene (within CNVR_59, common to all breeds), participate in a wide range of physiological processes, including the regulation of lipid metabolism, neurodevelopment, and transport of nutrients and vitamins [52,53], but also in cell proliferation process, making it a potential candidate gene for the supernumerary nipple phenotype [54]. The *KHDRBS2* gene, within the CNVR_310 with the highest variance, has been associated with fertility and reproductive traits in cattle [55], goat [56], and sheep [57]. Moreover, the *FILIP1* gene (CNVR_185), reported by Salehian-Dehkordi et al. [57] has been linked with fertility traits. The *ACAD9* gene is a recently identified acyl-CoA dehydrogenase that demonstrates maximum activity with unsaturated long-chain acyl-CoAs [58]. We found several genes involved in lipid metabolism (*AGMO*, *PNPLA2*, *SIRT3*, *KLF15*, *MGLL*, *ACAD9,* and *COPG1*) [59,60,61,62,63] and immune response (*ANO9*, *SIGGIR*, *PKP3*, *STIM1*, *PLXNA1*, *IFITM3*, *IFITN5*, *NNT*, *CREB1,* and *IRF7*) [64,65,66,67,68,69].

The genes *LOC101123149* and *LOC101123408* are olfactory receptors. Olfactory receptors are interesting candidates for physiological requirements of domestic animals [70] and for feed efficiency, as their expression has been detected in the gut and may be related to feed intake. Olfactory receptors in the gut may serve as sensors of chemical or nutritional status and may have a role in nutrient absorption or digestive function [71].

Finally, two candidate genes—*TBL1XR1* and *NNT*—mapped within the CNVRs were identified only in Barbaresca breed. *TBL1XR1* has been reported as a candidate gene for backfat thickness in cattle [72], and *NNT* as a candidate gene related with heat stress response [73]. Among the five breeds involved in this study, the Barbaresca is the only sheep with fat tail [30,31]. The fat tail is considered an adaptive response of animals to a hazardous environment and for facing future climate changes. Fat depots act as an energy reserve that allows sheep to survive extreme environments and conditions such as prolonged droughts, cold, and food scarcity [74,75,76,77]. Therefore, these genes are consistent with the phenotypic characteristics of the breed.

All these aforementioned genesbelong to CNVR loss, while only *FILIP1* gene is found in a CNVR gain.

## 5. Conclusions

In this work, we detected the CNVs and CNVRs in five sheep breeds and performed a CNV-based population genetics analysis. The present study provides a broader CNV map and is the first population genetic analysis in Italian sheep breeds conducted using CNVs. The number of CNVRs identified is not directly comparable with others previously reported due to technical differences in the methods used. Our results indicated that segregating CNVs expresses a certain degree of diversity across all breeds. CNV genetic markers may not be compatible with current population analyses, because they violate the classical population genetics assumptions based on the infinite allele model and the infinite site model for SNP. However, despite the low genetic differentiation among the five sheep breeds involved in this study, the clustering methods based on CNVRs arranged groups according to the breed they belong to. Therefore, our results corroborated previous studies and showed that CNVs represent a valuable molecular resource for providing good information for separating the population and could be further used to explore the function and evolutionary aspects of sheep genome.

## Figures and Tables

**Table 1:** Summary of CNVs identified in each breed.

Breed	N. Sample	N. CNVs	CNVs per Sample Min–Max (Average)	Loss	Gain	Min Length (bp)	Max Length (bp)	Mean Length (bp)
BARB	19	431	15–33 (23)	328	103	19,028	2,499,938	222,990
COM	43	1172	18–39 (27)	957	215	19,041	3,660,245	344,790
PIN	47	1216	11–43 (26)	963	253	23,587	4,399,691	399,121
SAR	24	481	12–28 (20)	335	146	23,587	3,692,295	272,509
VDB	294	6907	11–52 (24)	5469	1438	13,128	14,995,713	569,560
Total	427	10,207	11–52 (24)	8052	2155	13,128	14,995,713	1,808,970

Barbaresca (BARB), Comisana (COM), Pinzirita (PIN), Sarda (SAR), and Valle del Belìce (VDB).

**Table 2:** Summary of CNVRs identified in each breed.

Breed	N. CNVRs	Loss	Gain	Min Length (bp)	Max Length (bp)	Mean Length (bp)
BARB	83	61	22	42,405	2,013,519	178,021
COM	195	159	36	19,322	3,295,789	324,599
PIN	186	147	39	23,587	2,962,879	347,976
SAR	89	61	28	43,456	1,954,981	196,644
VDB	687	532	155	14,264	11,305,268	434,543
Total	1240	960	280	14,264	11,305,268	1,481,783

Barbaresca (BARB), Comisana (COM), Pinzirita (PIN), Sarda (SAR), and Valle del Belìce (VDB).

**Table 3:** Pairwise PhiPT genetic distances among the five breeds.

	BARB	COM	PIN	SAR	VDB
BARB	0.000				
COM	0.255	0.000			
PIN	0.264	0.077	0.000		
SAR	0.220	0.196	0.203	0.000	
VDB	0.097	0.126	0.114	0.084	0.000

Barbaresca (BARB), Comisana (COM), Pinzirita (PIN), Sarda (SAR), and Valle del Belìce (VDB).

**Table 4:** The gene ontology (GO) in the CNVRs identified in the five sheep breeds.

**Accession Number**	**Biological Process**	**Gene Symbol**
GO:0065007	Biological regulation	*DEAF1*, *HRAS*, *HNRNPF*, *PIDD1*, *TFAP2D*, *EPS8L2*, *EEFSEC*, *PAIP1*, *PNPLA2*, *KLF15*, *EPHB3*, *TXNRD3*, *ZXDC*, *FXYD4*, *CNBP*, *PLXNA1*, *GATA2*, *KHDRBS2*, *RUVBL1*, *CREB1*, *PDGFA*, *STIM1*, *IRF7*, *SYT4*, *SENP6*, *STIM1*, *TBL1XR1*
GO:0009987	Cellular process	*DEAF1*, *PSMD13*, *HRAS*, *EFCC1*, *HNRNPF*, *SUN1*, *PIDD1*, *TFAP2D*, *EPS8L2*, *MCM2*, *EEFSEC*, *MYO6*, *CHCHD6*, *METTL21A*, *NTNG2*, *PAIP1*, *PNPLA2*, *TMEM80*, *NNT*, *KLF15*, *EPHB3*, *FAM20C*, *SEC61A1*, *PKP3*, *GET4*, *SLC25A22*, *RPN1*, *RRM1*, *PKP3*, *TXNRD*, *ZXDC*, *RAB7A*, *ISY1*, *COPG1*, *FXYD4*, *RTKN2*, *CNBP*, *PLXNA1*, *GATA2*, *KHDRBS2*, *MYO6*, *RUVBL1*, *ANO9*, *CREB1*, *PDGFA*, *CHCHD6*, *STIM1*, *INTS1*, *IRF7*, *SYT4*, *SENP6*, *STIM1*, *SHROOM3*, *TBL1XR1*, *EFCC1*, *IQSEC1*
GO:0008152	Metabolic process	*DEAF1*, *PSMD13*, *EFCC1*, *HNRNPF*, *PIDD1*, *TFAP2D*, *MCM2*, *EEFSEC*, *METTL21A*, *PAIP1*, *PNPLA2*, *NNT*, *KLF15*, *EPHB3*, *FAM20C*, *SEC61A1*, *SLC25A22*, *RPN1*, *RRM1*, *ZXDC*, *METTL21A*, *ISY1*, *CNBP*, *GATA2*, *KHDRBS2*, *RUVBL1*, *CREB1*, *PDGFA*, *INTS1*, *IRF7*
GO:0050896	Response to stimulus	*HRAS*, *PIDD1*, *EPS8L2*, *NLRP6*, *MCM2*, *CHCHD6*, *NTNG2*, *EPHB3*, *PLXNA1*, *PDGFA*, *CHCHD6*, *SYT4*
GO:0002376	Immune system process	*ANO9*, *SIGGIR*, *PKP3*, *STIM1*, *PLXNA1*, *IFITM3*, *IFITN5*, *NNT*, *CREB1*, *IRF7*
**Accession number**	**Molecular function**	**Gene Symbol**
GO:0005488	Binding	*DEAF1*, *HRAS*, *EFCC1*, *HNRNPF*, *SUN1*, *TFAP2D*, *EPS8L2*, *MCM2*, *EEFSEC*, *MYO6*, *PAIP1*, *NNT*, *ACAD9*, *KLF15*, *SEC61A1*, *PKP3*, *RRM1*, *PKP3*, *RTKN2*, *CNBP*, *GATA2*, *KHDRBS2*, *MYO6*, *CREB1*, *PDGFA*, *STIM1*, *IRF7*, *POLR2L*, *SYT4*, *STIM1*, *SHROOM3*, *EFCC1*
GO:0003824	Catalytic activity	*HRAS*, *PIDD1*, *MGLL*, *MCM2*, *MYO6*, *METTL21A*, *PNPLA2*, *B4GALNT4*, *NNT*, *ACAD9*, *EPHB3*, *FAM20C*, *ALDH1L1*, *RRM1*, *TXNRD3*, *MYO6*, *RUVBL1*, *POLR2L*, *SENP6*
GO:0098772	Molecular function regulator	*DEAF1*, *EFCC1*, *TFAP2D*, *EPS8L2*, *KLF15*, *ZXDC*, *FXYD4*, *GATA2*, *CREB1*, *STIM1*, *IRF7*, *TBL1XR1*
GO:0005215	Trasporter activity	*SEC61A1*, *SLC25A22*, *FXYD4*, *ANO9*, *STIM1*
**Accession number**	**Cellular Component**	**Gene Symbol**
GO:0110165	Cellular anatomical entity	*DEAF1*, *PSMD13*, *ABTB1*, *HRAS*, *EFCC1*, *HNRNPF*, *SUN1*, *PIDD1*, *MGLL*, *LRP1B*, *STBD1*, *TFAP2D*, *EPS8L2*, *MCM2*, *MYO6*, *CHCHD6*, *METTL21A*, *NTNG2*, *PNPLA2*, *TMEM80*, *NNT*, *ACAD9*, *KLF15*, *EPHB3*, *FAM20C*, *SEC61A1*, *PKP3*, *GET4*, *RPN1*, *RRM1*, *TXNRD3*, *NUP210*, *ZXDC*, *CD151*, *RAB7A*, *RAB43*, *ISY1*, *COPG1*, *RTKN2*, *CNBP*, *IFITM5*, *PLXNA1*, *GATA2*, *KHDRBS2*, *RUVBL1*, *ANO9*, *CREB1*, *PDGFA*, *SLC41A3*, *INTS1*, *IRF7*, *SYT4*, *SENP6*, *STIM1*, *SHROOM3*, *TBL1XR1*, *ADAP1*
GO:0005622	Intracellular	*DEAF1*, *PSMD13*, *ABTB1*, *EFCC1*, *HNRNPF*, *SUN1*, *PIDD1*, *TFAP2D*, *MCM2*, *MYO6*, *CHCHD6*, *METTL21A*, *PNPLA2*, *NNT*, *ACAD9*, *KLF15*, *FAM20C*, *SEC61A1*, *PKP3*, *GET4*, *RPN1*, *RRM1*, *TXNRD3*, *NUP210*, *ZXDC*, *RAB7A*, *RAB43*, *ISY1*, *COPG1*, *RTKN2*, *CNBP*, *GATA2*, *KHDRBS2*, *RUVBL1*, *CREB1*, *STIM1*, *INTS1*, *IRF7*, *SYT4*, *SENP6*, *SHROOM3*, *TBL1XR1*, *ADAP1*
GO:0032991	Protein-containing complex	*PSMD13*, *ABTB1*, *HNRNPF*, *SUN1*, *MCM2*, *TMEM80*, *EPHB3*, *SEC61A1*, *GET4*, *RPN1*, *RRM1*, *NUP210*, *ISY1*, *COPG1*, *PLXNA1*, *RUVBL1*, *CREB1*, *INTS1*, *TBL1XR1*

## Data Availability

The data that support the findings of this study are available on request from the corresponding author.

## Supplementary Materials

The following are available online at https://www.mdpi.com/article/10.3390/ani12020217/s1. Table S1: CNVs identified in the Sicilian sheep breeds. Table S2: CNVRs identified in Sicilian sheep breeds. Table S3: Genes content within the top 10% of CNVRs based on their variances. Table S4: Analysis of molecular variance (AMOVA) showing the partitioning of genetic variation within and between breeds.
